# Tobacco Usage and Its Association With Mental Health Status of School-Going Adolescents Near Patna, Bihar: A Community-Based Cross-Sectional Study in Eastern India

**DOI:** 10.7759/cureus.39033

**Published:** 2023-05-15

**Authors:** Ria Roy, Pankaj Kumar, Sanjay Pandey, Alok Ranjan, Chandramani Singh

**Affiliations:** 1 Community and Family Medicine, All India Institute of Medical Sciences, Patna, Patna, IND; 2 Psychiatry, All India Institute of Medical Sciences, Patna, Patna, IND

**Keywords:** strengths and difficulties questionnaire, mental illness, smoking, tobacco, mental health

## Abstract

Background

Of all the adolescents in India, 7.3% are suffering from some form of mental disorder. They frequently use tobacco to cope with these problems, but get stuck in a vicious cycle of deteriorating mental health. Our study aimed to determine the effect of tobacco on the mental health status of adolescents studying in the 9th to 12th standards in 10 high schools in urban and rural areas near Patna, Bihar.

Methodology

An analytical cross-sectional study was conducted among 360 school-going adolescents recruited using stratified random sampling. Selected adolescents were given the Indian Adolescent Health Questionnaire. The mental health status was calculated from the Strengths and Difficulties Questionnaire (SDQ) score. Information on sociodemographic characteristics and tobacco use was also obtained. Independent t-test, analysis of variance, and multiple linear regression analysis were used for predicting the significant factors. Significance was set at p-values <0.05.

Results

In this study, 40 (11.1%) adolescents had abnormal whereas 55 (15.3%) had borderline overall SDQ scores. The majority of those affected had peer problems (40%) and conduct problems (24.7%). All SDQ components of conduct (F = 2.94, p = 0.013), hyperactivity (F = 2.90, p = 0.014), emotional problems (F = 1.14, p = 0.001), and peer pressure (F = 3.06, p = 0.010), as well as the overall SDQ score (F = 5.74, p < 0.001), were significantly associated with increasing age. The adolescents attending rural schools (13.28 ± 5.22, p = 0.047) had significantly higher SDQ scores than those attending urban schools (12.08 ± 5.60). Hyperactivity scores were significantly higher in those studying in class 10 compared to other classes and in those attending rural rather than urban schools. Emotional problems score was significantly higher in 16-17-year-old students than in 14-15-year-old students, in females than in males, and in class 10 than in class 9 students. Only 24 (6.7%) adolescents had a history of tobacco consumption at least once which was significantly associated with the SDQ score (17.71 ± 5.69; t = 4.95, df = 358, p < 0.001). Around 79.4% of adolescents were exposed to passive smoking from close friends which deteriorated their overall mental health status (14.50 ± 5.99; F = 6.29, df = 2,357, p = 0.002). Those who had smoked for more than 10 days had significantly greater conduct problems and lesser pro-social behavior. Overall, 96.1% agreed that tobacco is harmful to health, and 76.1% had seen anti-smoking messages in the media. Female gender, increasing class and age, and history of smoking or chewing tobacco at least once also led to a significant increase in emotional problems. Age, area of the school, history of tobacco consumption, and exposure to cigarette smoke by either a close friend or male guardian had a significant impact on the conduct, hyperactivity, peer problems, and overall mental health status of school-going adolescents.

Conclusions

Predicting risk factors such as age, area of the school, and the history of tobacco consumption by self or by close friends is important for decision-making by school administration regarding counseling for mental health and preventing tobacco use.

## Introduction

Adolescents are defined as people aged 10-19 years. A recent Lancet commission divided adolescence into two categories, namely early (10-14 years) and late (15-19 years) adolescence [1]. In the past, adolescent health had been neglected because they were thought to be less vulnerable than other age groups [2]. Adolescence is a unique formative period when many physical, emotional, and social changes, including exposure to undesirable situations, risk factors, and lifestyles, can make them vulnerable to mental health problems. Of the world’s 1.22 billion adolescents, a staggering majority of one-fifth, i.e., 253.2 million, live in India. Among them, 7.3% are suffering from some form of mental disorder, with a similar prevalence in males and females [3]. The psychological morbidity of the adolescent age group can be due to the occupation of their parents, the expectation of high achievements, academic pressure, peer influences, and poor socioeconomic conditions. Gender, religion, class of study, and home environment can cause depressive symptoms; yet, 70% of adolescents do not seek any help or treatment [4]. They frequently use tobacco to cope with these problems, but get stuck in a vicious cycle of deteriorating mental health. The use of tobacco and other harmful substances is gradually increasing among the school-going population across all socioeconomic strata [5].

Much research has been done on the prevalence of mental health problems in different parts of India, with several studies focusing on children and adolescents. However, very few studies have investigated potential risk factors such as the harmful use of tobacco in school-going adolescents, leading to mental health problems. The determinants for mental health problems and their association with tobacco usage were not researched previously in this population of Bihar, eastern India. Hence, this study aimed to determine the effect of tobacco on the mental health status of adolescents studying in the 9th to 12th standards in the rural and urban areas near Patna, Bihar.

## Materials and methods

Study design and duration

An analytical cross-sectional study was conducted among school-going adolescents studying in the 9th to 12th standards in 10 high schools located in rural and urban areas near Patna, Bihar. The study was conducted from September 2020 to February 2021.

Study participants

Inclusion Criteria

In each school, there were two mandatory visits and an optional third visit. On the first day, students were selected and given informed assent forms, along with consent forms (to obtain consent from their guardians). Students who brought back the signed consent forms on the second day were enrolled in the study. These students were asked to fill out the self-administered Indian Adolescent Health Questionnaire (IAHQ) [6]. If more than five selected students did not bring the consent forms or were absent on the second day, then an optional third visit was done to include the students.

Exclusion Criteria

If the selected students were absent on both the second and third days, they were excluded. Additionally, the selected students were asked on the first day if they were diagnosed with depression/anxiety/sleep disorders. If any student was found to suffer from these problems, he/she was excluded and another student was selected instead for the study.

Sampling technique

We used the stratified random sampling technique. First, we stratified all schools based on the urban and rural practice areas of the Department of Community and Family Medicine, All India Institute of Medical Sciences, Patna. A total of 13 schools were stratified, and 10 schools (six urban and four rural schools) were randomly chosen with a proportional allocation method. Thereafter, the students in each school were randomly selected from each of the classes from 9th to 12th, again with a proportional allocation method.

Sample size calculation

The sample size was estimated using Cochran’s formula for estimation of prevalence using the following formula: n = [Z 2p( 1 - p )]/d2.

Taking the prevalence of mental health disorders among school-going adolescents from a meta-analysis by Malhotra and Patra [7], the p-value was considered to be 23.3%. The margin of error or d was taken as a relative p-value, i.e., 20% of p, which is 4.66. The sample size came out as 320. Taking a margin of 10% for lost data, n = 320 × 100/90 = 355. After rounding off the value, the final sample size n is 360. The number of schools was 10. Hence, the sample size required from each school was 360/10 = 36.

Study tool and procedure

The IAHQ [6] is a user-friendly, school-based, linked, anonymous, comprehensive health assessment tool, which has been previously tested and validated. Oxford University Press license for IAHQ was taken online before the commencement of the study. Students could answer the questionnaire in either English or Hindi. The instructions for filling out the questionnaire were explained to the students, and approximately 30 minutes were given for filling out the forms. Each student filled out the form separately from others to avoid the bandwagon effect.

There are a total of 12 domains in the IAHQ, out of which for our study purpose we considered the domains of mental health (Strengths and Difficulties Questionnaire (SDQ)), cigarette and tobacco use, and sociodemographic domain consisting of age, gender, type of the school, area of the school, the standard they are in, and type of family. Under cigarette and tobacco Use, there are six questions, as shown in Table 1.

**Table 1:** Cigarette and tobacco use questions in the Indian Adolescent Health Questionnaire.

Cigarette and tobacco use	Options
Have you ever smoked cigarettes/chewed tobacco?	Never tried/Tried once
If yes, how many days did you smoke/chew tobacco?	<10 days/10 days or more
Have any of your close friends ever tried smoking cigarettes or chewing tobacco?	Yes/Unsure/No
Which of your parents or guardians smoke?	Neither/Father or male guardian/Mother or female guardian/Both
Do you think smoking is harmful to your health?	Yes/Unsure/No
In the past month, have you seen any anti-smoking media messages (on television, radio, billboards, posters, newspapers, magazines, and movies)?	Yes/Unsure/No

The SDQ [8] consists of 25 questions that refer to different emotions or behaviors in the last six months, divided into five scales each containing five questions (Table 2). The scales include emotional symptoms, conduct problems, hyperactivity/inattention, peer relationship problems, and pro-social behavior (reverse coded). The responses to the questions are graded on a Likert scale of 3 as *Never in 6 months* (score 0), *Between 1 and 10 times in 6 months* (score 1), and *More than 10 times in 6 months* (score 2). Then the scores for the first four scales only were summed to generate an overall total SDQ score out of a maximum score of 40. The overall SDQ score was normal at a score of 0-15, borderline at a score of 16-19, or abnormal at a score of 20-40.

**Table 2:** Strengths and Difficulties Questionnaire and Scoring [8]

Scale	Questions/Statements	Normal score	Borderline score	Abnormal score
Conduct score	I get very angry and often lose my temper	0–3	4	5–10
	I usually do as I am told			
	I fight a lot. I can make other people do what I want			
	I am often accused of lying or cheating			
	I take things that are not mine from home, school, or elsewhere			
Hyperactivity score	I am restless, I cannot stay still for long	0–5	6	7–10
	I am constantly fidgeting or squirming			
	I am easily distracted. I find it difficult to concentrate			
	I think before I do things			
	I finish the work I’m doing. My attention is good.			
Emotional problems score	I get a lot of headaches, stomachaches, and sickness	0–5	6	7–10
	I worry a lot			
	I am often unhappy, depressed, or tearful			
	I am nervous in new situations. I easily lose confidence			
	I have many fears, and I am easily scared			
Peer problems score	I would rather be alone than with people of my age	0–3	4–5	6–10
	I have one good friend or more			
	Other people my age generally like me			
	Other children or young people pick on me or bully me			
	I get along better with adults than with people my own age			
Pro-social behavior score	I try to be nice to other people. I care about their feelings	6–10	5	0–4
	I usually share readily with others (treats, toys, pencils, etc.)			
	I am helpful if someone is hurt, upset, or feeling ill			
	I am kind to younger children			
	I often offer to help others (parents, teachers, and other children)			

Data analysis

The forms were collected and data were entered into Microsoft Excel 2019. Then the data were imported into SPSS version 22 (IBM Corp., Armonk, NY, USA). The dependent variable, i.e., mental health status, was calculated based on the SDQ score from the responses of the participants. The independent variables taken for the study were sociodemographic characteristics and cigarette and tobacco use. The overall SDQ score along with the score of its component scales and pro-social behavior score were analyzed across these independent variables using an independent t-test or analysis of variance (ANOVA). Multiple linear regression analysis was used to predict the significant factors. The regression model was tested for multicollinearity, homoscedasticity, linearity, and normality. The significance level was set at a p-value <0.05.

Ethical consideration

There was no risk to the study participants, and approval for the study was obtained from the Institutional Ethics Committee of All India Institute of Medical Sciences, Patna on November 05, 2019 (reference number: AIIMS/Pat/IEC/PGTh/Jan19/07). The selected students were given informed written assent forms along with consent forms to take informed written consent from their guardians for enrolment into the study.

## Results

A total of 360 adolescents studying in the 9th to 12th standards in private (88.9%) and government (11.1%) schools located in rural (36%) and urban (64%) areas of the field practice area of the Department of Community and Family medicine, AIIMS, Patna were enrolled in the study. The male-to-female ratio of the adolescents in the study was 1.6. Table 3 presents the distribution of the overall SDQ and its component scale scores. In total, 40 (11.1%) adolescents had abnormal whereas 55 (15.3%) had borderline SDQ scores. Thus, more than a quarter of adolescents (26.4%) had non-normal SDQ scores (16-40), but the overall mean score was 12.51 ± 5.49. Among individual mental health scales, adolescents mainly had peer problems (abnormal = 10% and borderline = 30%, mean score = 3.15 ± 1.66) and conduct problems (abnormal = 11.9% and borderline = 12.8%, mean score = 2.52 ± 1.78). Similarly, 13.3% had abnormal and 8.9% had borderline pro-social behavior scores (mean score = 7.09 ± 2.18).

**Table 3:** Distribution of the SDQ score (n = 360). SDQ = Strengths and Difficulties Questionnaire

	Conduct (n, %)	Hyperactivity (n, %)	Emotional (n, %)	Peer problems (n, %)	Pro-social behavior (n, %)	Overall SDQ (n, %)
Normal	271, 75.3%	309, 85.8%	296, 82.2%	216, 60.0%	280, 77.8%	265, 73.6%
Borderline	46, 12.8%	29, 8.1%	24, 6.7%	108, 30.0%	32, 8.9%	55, 15.3%
Abnormal	43, 11.9%	22, 6.1%	40, 11.1%	36, 10.0%	48, 13.3%	40, 11.1%
Mean ± SD	2.52 ± 1.78	3.45 ± 1.89	3.39 ± 2.28	3.15 ± 1.66	7.09 ± 2.18	12.51 ± 5.49

Table 4 presents the association of the SDQ score and its components with the sociodemographic characteristics of the participants. All SDQ components of conduct (F = 2.94; df = 5,354; p = 0.013), hyperactivity (F = 2.90; df = 5,354; p = 0.014), emotional problems (F = 1.14; df = 5,354; p = 0.001), and peer pressure (f = 3.06; df = 5,354; p = 0.010), as well as the overall SDQ score (F = 5.74; df = 5,354; p < 0.001), were significantly associated with increasing age, as seen by ANOVA. Conduct problems were significantly different between ages 13 (1.74 ± 1.48) and 17 years (3.29 ± 2.09, Bonferroni post-hoc p = 0.048). Emotional problems score was significantly more in 16-17-year-old students (score = 3.87-4.75) than in 14-15-year-old students (score = 2.85-3.27), in females (3.96 ± 2.25, p < 0.001) than in males (3.03 ± 2.23), and in class 10 (3.87 ± 2.23, p < 0.001) than in class 9 (2.87 ± 2.22) students. However, class 10 students were found to be more pro-social (7.40 ± 2.10, p = 0.034) than students of other classes, especially class 9 (6.77 ± 2.24). The SDQ score was significantly higher in 16-17-year-old (score = 14.15-16.04) than in 13-15-year-old students (score = 10.58-12.02). Around 230 (63.9%) students were attending schools in urban areas, and 130 (36%) students in rural areas. Hyperactivity scores were found to be significantly higher in those studying in class 10 (3.76 ± 1.89, p = 0.044) compared to other classes, and in those attending rural schools (3.75 ± 1.87, p = 0.025) rather than in urban schools (3.28 ± 1.89). The adolescents attending rural schools (13.28 ± 5.22, p = 0.047) had significantly higher overall SDQ scores than those attending urban schools (12.08 ± 5.60). The SDQ score was also associated with classes (F = 4.15; df = 3,356; p = 0.007), especially between class 10 (13.62 ± 5.4) and class 9 students (11.52 ± 5.4, Bonferroni post-hoc p = 0.003).

**Table 4:** Association of the sociodemographic characteristics of the participants with different component scores and the overall SDQ score (n = 360). The table shows the association of the different sociodemographic characteristics with each component of the SDQ and the overall SDQ score. The association has been calculated by comparing the categories of each of the sociodemographic variables using *independent t-test analysis and **analysis of variance tests. The p-values in bold represent statistically significant differences (p < 0.05). SDQ = Strengths and Difficulties Questionnaire

Sociodemographic characteristics	Conduct (p-value)	Hyperactivity (p-value)	Emotional (p-value)	Peer problems (p-value)	Pro-social behavior (p-value)	Overall SDQ (p-value)
Age (in years)**						
13	0.013	0.014	0.001	0.010	0.639	<0.001
14						
15						
16						
17						
18						
Gender*						
Male	0.821	0.494	<0.001	0.558	0.061	0.154
Female						
Type of school*						
Government	0.409	0.063	0.155	0.687	0.772	0.278
Private						
Area of school*						
Rural	0.079	0.025	0.529	0.198	0.852	0.047
Urban						
Standard**						
9	0.329	0.044	<0.001	0.718	0.034	0.007
10						
11						
12						
Type of family*						
Joint	0.404	0.591	0.879	0.656	0.392	0.876
Nuclear						

Table 5 presents the association of a history of tobacco chewing or cigarette smoking. In total, 24 (6.7%) adolescents had chewed or smoked tobacco at least once, and they had significantly high conduct (4.04 ± 1.46; t = 4.452; df = 358; p < 0.001), hyperactivity (4.79 ± 1.91; t = 3.65; df = 358; p < 0.001), emotional (4.58 ± 2.84; t = 2.67; df = 358; p = 0.008), peer problems (4.29 ± 1.76; t = 3.55; df = 358; p < 0.001), and overall SDQ (17.71 ± 5.69; t = 4.95; df = 358; p < 0.001) scores. Among the 24 adolescents, only five (20.8%) had used tobacco for 10 days or more in the previous month. It had no association with the overall SDQ score but they showed significantly greater conduct problems (5.60 ± 1.67; t = 3.17; df = 22; p = 0.004) and lesser pro-social behavior (4.60 ± 7.32; t = -2.40; df = 22; p = 0.025) scores. Conduct problems (3.18 ± 1.93; F = 6.72; df = 2,357; p < 0.001), hyperactivity (4.12 ± 2.03; F = 6.03; df = 2,357; p = 0.003), and overall SDQ score (14.50 ± 5.99; F = 6.29; df = 2,357; p = 0.002) were higher in 79.4% of adolescents who were exposed to their friends consuming tobacco. Only 49 (13.6%) of them were exposed to smoking by their father or male guardian, and they had significantly high hyperactivity scores (4.29 ± 2.0; t = 3.37; df = 358; p = 0.001) than those who were not exposed through family (3.32 ± 1.85). However, there was a general awareness about the pitfalls of tobacco use. Overall, 96.1% of adolescents agreed that tobacco is harmful to health. Conduct score was significantly higher in those who did not think smoking was harmful to health (3.89 ± 1.36; F = 3.54; df = 2,357; p = 0.033). Again, 76.1% had seen anti-smoking messages in the media, but it had no significant association with any SDQ score.

**Table 5:** Association of a history of tobacco use and exposure of the participants with different component scores and the overall SDQ score. The table shows the association of the detailed history of tobacco use and exposure with each component of the SDQ and the overall SDQ score. The association has been calculated by comparing the categories of each variable using *independent t-test analysis and **analysis of variance tests. The p-values in bold represent statistically significant differences (p < 0.05). SDQ = Strengths and Difficulties Questionnaire

	Conduct (p-value)	Hyperactivity (p-value)	Emotional (p-value)	Peer pressure (p-value)	Pro-social behavior (p-value)	Overall SDQ (p-value)
History of cigarette smoking/chewing tobacco ever* (n = 360)						
Never tried	<0.001	<0.001	0.008	<0.001	0.424	<0.001
- Tried once						
Frequency of cigarette smoking/chewing tobacco in the past month* (n = 24)						
Fewer than 10 days	0.004	0.992	0.483	0.059	0.025	0.104
10 days or more						
Exposure to cigarette smoking by close friend** (n = 360)						
Yes	0.001	0.003	0.183	0.354	0.223	0.002
Unsure						
No						
Exposure to cigarette smoking by either parent* (n = 360)						
Never	0.130	0.001	0.426	0.688	0.384	0.065
Father/male guardian						
Opinion on whether tobacco is harmful to health** (n = 360)						
Yes	0.033	0.519	0.920	0.989	0.197	0.378
Unsure						
No						
Whether seen any anti-smoking media messages** (n = 360)						
Yes	0.835	0.868	0.569	0.196	0.725	0.884
Unsure						
No						

Table 6 presents the final multiple linear regression models predicting each of the mental health scores. Increasing age, rural area schools, a history of cigarette smoking or tobacco chewing at least once, and a history of tobacco consumption by a close friend significantly increased the overall SDQ score of school-going adolescents. However, these four predictors were only able to explain 12.8% of the variance in the SDQ score (F = 14.139, p < 0.001). Again, increasing age, history of tobacco consumption by a close friend, and opinion that smoking does not harm health contributed to the increase in conduct problems (F = 8.605, p < 0.001, adj. R^2 ^= 6%). History of tobacco consumption by father or male guardian, alongside increasing age, rural area schools, and a history of tobacco consumption at least once, predicted a significant increase in hyperactivity in the adolescents (F = 9.924, p < 0.001, adj. R^2^ = 9%). Female gender, increasing class and age, and a history of smoking or chewing tobacco at least once also led to a significant increase in emotional problems (F = 11.818, p < 0.001, adj. R^2^ = 10.8%). Finally, only increasing age and history of tobacco consumption at least once predicted a significant increase in the peer problems score (F = 11.190, p < 0.001, adj. R^2^ = 5.4%).

**Table 6:** Multiple linear regression models predicting significant factors leading to mental health morbidity (n = 360). SDQ = Strengths and Difficulties Questionnaire

Predictors	B (95% CI)	P-value	F (p-value)	R^2^ (Adj. R^2^)
Conduct problems score				
(Increasing) Age	0.240 (0.069, 0.411)	0.006	8.605 (<0.001)	6.8 (6.0)
Exposure to cigarette smoking by a close friend (Yes)	0.363 (0.139, 0.587)	0.002		
Opinion on whether smoking is harmful to health (Yes)	-0.628 (-1.169, -0.086)	0.023		
Constant	-0.096 (-2.929, 2.737)	0.947		
Hyperactivity score				
(Increasing) Age	0.312 (0.131, 0.493)	0.001	9.924 (<0.001)	10.1 (9.0)
Area of school (Urban)	-0.480 (-0.871, -0.088)	0.017		
History of cigarette smoking/chewing tobacco ever (Tried once)	0.899 (0.280, 1.518)	0.005		
Exposure to cigarette smoking by father/male guardian	0.888 (0.339, 1.436)	0.002		
Constant	-2.010 (-4.704, 0.685)	0.143		
Emotional problems score				
Increasing (Age)	0.299 (0.039, 0.558)	0.024	11.818 (<0.001)	11.8 (10.8)
Gender (Female)	1.072 (0.607, 1.536)	<0.001		
(Increasing) Class	0.358 (0.002, 0.714)	0.049		
History of cigarette smoking/ chewing tobacco ever (Tried once)	1.054 (0.310, 1.798)	0.006		
Constant	-6.092 (-9.583, -2.601)	0.001		
Peer problems score				
Increasing (Age)	0.203 (0.042, 0.365)	0.014	11.190 (<0.001)	5.9 (5.4)
History of cigarette smoking/chewing tobacco ever (Tried once)	1.010 (0.457, 1.562)	<0.001		
Constant	-0.990 (-3.396, 1.416)	0.419		
Overall SDQ score				
(Increasing) Age	1.141 (0.627, 1.655)	<0.001	14.139 (<0.001)	13.7 (12.8)
Area of school (Urban)	-1.463 (-2.574, -0.353)	0.01		
History of cigarette smoking/chewing tobacco ever (Tried once)	4.287 (2.049, 6.526)	<0.001		
Exposure to cigarette smoking by a close friend (Yes)	0.757 (0.072, 1.443)	0.031		
Constant	-8.555 (-16.291, -0.819)	0.030		

## Discussion

Over the years, there has been a gradual increase in substance use among all demographics of the young population (10-24 years). The average age of initiation of tobacco use was found to be the lowest among all substances. Familial risk factors such as similar substance use by one or more family members, conflicts, physical abuse, poverty, urbanization, broken families, and domestic violence contribute significantly to this growing phenomenon [5]. Coincidentally, these are the very same risk factors that lead to mental health disorders. The Global Youth Tobacco Survey-4 (GYTS-4) by the Indian Institute of Population Sciences in 2019 revealed that 8.5% of students used tobacco products, 7.3% smoked cigarettes, and 4.1% used the smokeless form. Tobacco use was higher among males. Around 29.5% of the students were exposed to second-hand smoke, and 52% of students had noticed anti-tobacco messages in the mass media [9]. Compared to this recent data, our study revealed that 6.7% had smoked or used tobacco at least once, with exposure to second-hand smoke from either the father or a male guardian (13.6%) or a close friend (79.4%). This is higher than the findings reported by the GYTS-4 survey. Moreover, the awareness among adolescents was quite high in our study, with 96.1% agreeing that tobacco is harmful to health, and 76.1% noticing anti-smoking messages in the media. Increased dissemination of information and behavioral change programs with counseling can eventually reduce the consumption of tobacco products, but for now, they have an impact on the mental health status of adolescents.

The SDQ is a tool used for measuring general psychopathology and associated impairment among children, adolescents, parents, or teachers [10]. Our study used the SDQ tool meant for adolescents. The psychometric properties of the scale harbor information from the subscales that indicate conduct problems, hyperactivity problems, emotional behavior, and behavior due to peer pressure. These subscales can indicate the extent of difficulties faced by adolescents [11]. According to a review of effective mental health interventions for young people in low and middle-income countries, schools are one of the most important community settings for promoting mental health among young people, leading to positive mental health, social, and educational outcomes. Having structure, delivery, and utilizing teachers and counselors can make the school-based approach more effective in handling mental health problems [6]. Interventions for psychosocial dysfunction need a robust start, targeting adolescents, their caregivers, and community stakeholders, especially with an emphasis on the school setting [12].

Most adolescents were experiencing peer problems and conduct problems. This can be attributed to academic underachievement, poor social skills and social competence, negative attitudes toward school, and lack of parental guidance. Peer affiliation can either be a negative or a positive influence on adolescents because they tend to fit in with their friends and behave in similar ways [13]. Age had a universal impact on all factors determining mental health. This is reflective of another study conducted by Cavioni et al. in Milan, Italy, where mental health was reported to be poorer in older adolescents (estimate = -0.050, p = 0.010) [14]. Even though emotional problems are not extensive, female adolescents are found to be more emotionally distraught than their male counterparts, probably due to the difference in their coping mechanisms in the face of peer pressure and school stress [15]. Adolescents going to schools in rural areas were more prone to mental health problems than those going to schools in urban areas. This is probably due to the latter being less impoverished, experiencing better education services, and having well-educated parents [16] who had a better understanding of their needs, who were involved in their educational activities, and who spent quality time with them [17]. Several studies have suggested that the level of mental illness and anxiety in rural areas had a higher impact than that experienced by children in urban areas [18,19]. Well-trained teachers who could relate and teach empathetically in private schools were important, along with the availability of more stress-relieving entertainment options in these areas. However, there is a risk of substance use, physical inactivity, and obesity in these adolescents compared to those from rural areas [20]. Students from classes 10 and 12, nearing their secondary and post-secondary examinations, respectively, experience a lot of stress. Major depressive episodes (MDEs) and substance use disorder are some highly concerning diseases in adolescence. Several studies show a nearly two-fold increase in MDE in the 13-14-year-old age group compared to the 17-18-year-old age group [21]. These mental health problems can be minimized by clinical interventions while supporting the transition to adulthood [22].

The most significant predictor for poor mental health in our study was a positive history of cigarette smoking or chewing tobacco. Globally, two out of three people with severe mental health conditions are current smokers, and they die almost 15-20 years prematurely. A study done by Caris et al. in Chile showed an overall positive association between the presence of behavioral problems and tobacco smoking among adolescent school students [23]. By reducing smoking among people with mental illness, we could take the single most effective action in reducing the gap in life expectancy [24]. Understanding the reason for the uptake of this habit is important as tobacco consumption leads to a long-term effect on the person’s mental health. Nicotine stimulates the release of dopamine in the brain, generating positive feelings. Dopamine is often low in people with depression, who then use cigarettes as a way of temporarily increasing their dopamine supply. However, smoking encourages the brain to turn off its own mechanism of making dopamine, so in the long term, the dopamine supply decreases, which, in turn, prompts people to smoke even more [25]. Conversely, quitting tobacco effectively can allow adolescents on psychiatric medications to reduce the dosage by up to 25% [24]. Having a close friend smoking in the vicinity creates an inducive environment for the uptake of smoking or any other habit related to tobacco use. Even exposure to others smoking around you can cause an increased incidence of mental health disorders, as seen in this study [26]. Another study among adolescent twins revealed that ever-smoking led to significantly more attention problems than never-smoking co-twins, lasting into adulthood [27]. There is also a risk factor for smoking or tobacco chewing in adolescents whose parents were more likely to be early regular smokers, and this risk increases with each additional year of exposure to parental smoking [28]. This is indirectly promoting smoking behavior among children, which has a highly significant association with inattention and hyperactive or impulsive symptoms [29,30]. This is their go-to response to prolonged psychological stress and can lead to the onset of physical and mental conditions. Given the potential mechanisms by which second-hand smoke exposure could promote or exacerbate mental health in adolescents, it is imperative to conduct further research investigating this association.

One of the strengths of this study is determining the self-reported perceptions regarding smoking, tobacco, and mental health issues. The use of a pre-validated questionnaire such as the IAHQ gives an accurate picture of the mental health status of adolescents. The study also points toward tobacco use and a few demographic factors as strong determinants of their mental health, resulting in long-term effects on these adolescents well into their adult life. However, significant demographic factors such as age and area of school could be confounding the results. There might also be information bias from the participants, as well as non-establishment of causal or risk factors in this cross-sectional study.

## Conclusions

Age, area of the school, history of tobacco consumption, and exposure to cigarette smoke by either a close friend or male guardian had a significant impact on the conduct, hyperactivity, peer problems, and overall mental health status of school-going adolescents. Female gender was also a significant predictor for high emotional problems in adolescents. Predicting these risk factors is important for decision-making by school administration regarding counseling about mental health and preventing tobacco use. Mental health problems are increasingly becoming an epidemic in India, hence, attention and intervention need to be given since adolescence to prevent the development of mental illnesses in the community.
